# Areal Surface Roughness Optimization of Maraging Steel Parts Produced by Hybrid Additive Manufacturing

**DOI:** 10.3390/ma13020418

**Published:** 2020-01-16

**Authors:** Philipp Wüst, André Edelmann, Ralf Hellmann

**Affiliations:** Applied Laser and Photonics Group, University of Applied Sciences Aschaffenburg, Würzburger Straße 45, 63743 Aschaffenburg, Germany; wuestphilipp@web.de (P.W.); ralf.hellmann@th-ab.de (R.H.)

**Keywords:** hybrid additive manufacturing, selective laser melting, ball end milling, maraging steel, design of experiments, Taguchi method, surface characterization

## Abstract

We report on an experimental study and statistical optimization of the surface roughness using design of experiments and the Taguchi method for parts made of 1.2709 maraging steel. We employ a hybrid additive manufacturing approach that combines additive manufacturing by selective laser melting with subtractive manufacturing using milling in an automated process within a single machine. Input parameters such as laser power, scan speed, and hatching distance have been varied in order to improve surface quality of unmachined surfaces. Cutting speed, feed per tooth, and radial depth of cut have been varied to optimize surface roughness of the milled surfaces. The surfaces of the samples were characterized using 3D profilometry. Scan speed was determined as the most important parameter for non-machined surfaces; radial depth of cut was found to be the most significant parameter for milled surfaces. Areal surface roughness $S_{a}$ could be reduced by up to 40% for unmachined samples and by 23% for milled samples as compared to the prior state of the art.

## 1. Introduction

Additive manufacturing (AM) or 3D printing is characterized by the principle of building components layer by layer, each layer representing a thin cross-section through the CAD data set [1]. In selective laser melting (SLM), a thin layer of metal powder is distributed across a build plate by a recoater and then selectively melted by a laser. The build plate is then lowered by the thickness of one layer and the process of recoating and melting is being repeated [2].

SLM parts usually have an unsatisfactory surface quality due to the high roughness. This limits the use of such components for applications with tight tolerances [3], high demands on fatigue strength [4], or where sterilizability in medical applications is required [5]. Subtractive post-processing, such as milling or turning, usually follows the additive manufacturing process for such components in order to meet the desired requirements. However, this additional work step involves further effort and equipment. A hybrid approach can overcome these issues by combining a non-traditional with a traditional process to obtain what, put in simplified terms, can be called a “1 + 1 = 3-effect” [6]: Freedom of design offered by additive manufacturing can be combined with the accuracy and surface quality of milling within a single, automated process. By combining additive and subtractive processes, components can comprise both machined and unmachined surfaces. Either way, it may be desirable to achieve the best possible surface finish. This requires both processes to be optimized. Against this background, this contribution focuses on hybrid additive manufacturing using SLM and three-axis milling. It is worthwhile to note that further hybrid AM processes, such as direct metal deposition and five axis milling, exist as discussed by Flynn et al. [3]. However, throughout this paper, we use the term hybrid AM as synonymous with selective laser melting combined with three-axis powder bed milling.

1.2709 or maraging steel 300 is a low-carbon and high-nickel steel. The term maraging refers to the fact that the material has a martensitic microstructure and that it can both be hardened and its strength be increased by aging. Due to its high strength, the alloy is used in tooling, structural, and aerospace applications [7]. Thus far, most of the publications on 1.2709 in additive manufacturing deal with density, porosity, mechanical properties, and microstructure in general [8,9], whereas only little research has been done regarding hybrid AM [10,11]. Additionally, work has been done on the machining of additively manufactured maraging steel, though not in a hybrid process [12].

When optimizing SLM and milling processes, a multitude of parameters emerge, each with a variety of factors. This usually results in a large number of experiments. Design of experiments (DoE) using Taguchi’s method narrows down the amount of experiments needed by using fractional factorial designs [13]. This method is widespread and common to achieve the best possible surface finish in milling processes [14] and has also been used for AM processes [15].

To the current state, there is only one study known to the authors on the surface roughness of milled hybrid AM parts [11], but there is no optimization towards the best possible surface quality for both machined and unmachined surfaces yet. Therefore, the aim of this paper is to show that Taguchi’s method is suitable for hybrid AM and to provide optimized parameters for components with machined and unmachined surfaces in order to obtain a low areal roughness.

## 2. Method

### 2.1. Experimental Procedure

#### 2.1.1. Machine and Process

**Figure 1:**
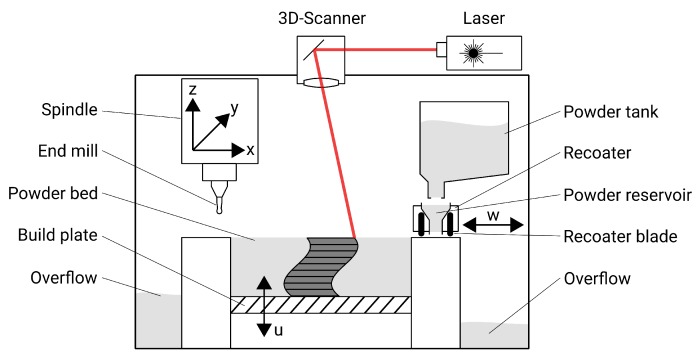
Schematic illustration including movable axes of the hybrid additive manufacturing (AM) machine used in this work.

In this study, a hybrid machine combining SLM and three-axis milling is used (*Matsuura LUMEX Avance-25*, Fukui, Japan). The schematic construction of such a machine is shown in Figure 1. The SLM part of the machine consists of a vertically movable buildplate onto which a thin layer of powder is applied by a recoating system and leveled with steel blades. Afterwards, a laser beam deflected by a 3D-scanner selectively melts the powder bed. Thus, the actual SLM process does not differ from the industry standard and represents the current state of the technology. The machine is equipped with a 400 W continuous wave Yb fiber laser (*SPI*, Southampton, UK) with a wavelength of 1070 nm. The maximum work size is 250×250 $mm^{2}$ in plane and 185 mm in height.

**Figure 2:**
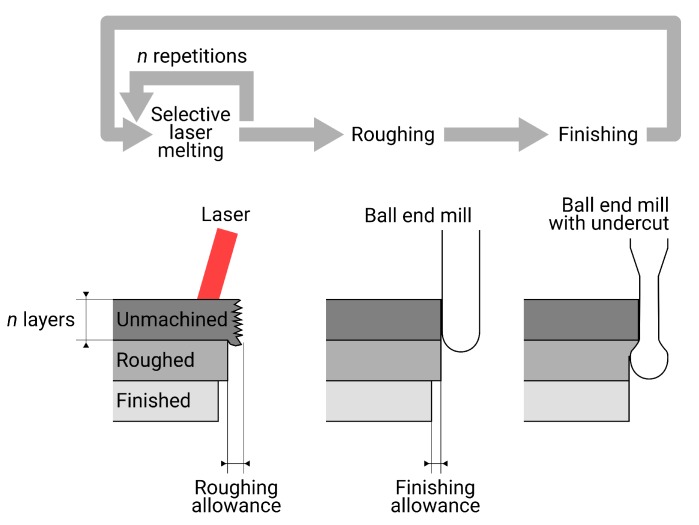
Sequence of a hybrid AM process consisting of selective laser melting and milling according to Nojiri et al. [16]

The fundamental working principle of the integrated milling machine does not differ from industry standards either. It incorporates a milling spindle with a maximum speed of 45,000 revolutions per minute. However, the uniqueness is the combination of the processes. After a certain number of SLM-built layers, usually ten layers with a height of 50 μm each, milling takes place. The contour respectively all outer surfaces are machined in a roughing and finishing operation. However, SLM and milling do not simply alternate because the thermal influence of the material build-up by the laser on the underlying layers is significant. Due to shrinkage during solidification and thermal residual stresses during cooling, distortion of the subjacent layers occurs. As a result, the dimensional accuracy of the precisely machined surfaces below is no longer maintained. To prevent this, the finishing process is thermally separated from SLM. This is done by finishing in a certain distance in the *z*-direction, e.g., 1 mm lower, from the last SLM-built layer [16]. Figure 2 shows the approach to separate the thermal influence of SLM from milling.

#### 2.1.2. Selective Laser Melting

**Figure 3:**
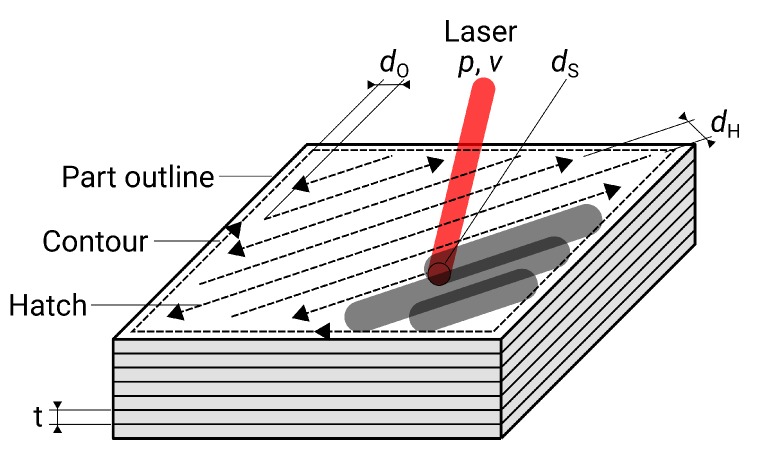
Exposure strategy and process parameters in selective laser melting (SLM).

There are two types of scanning tracks in SLM: The interior of the component is filled with alternating patterns, referred to as hatching. The outline of the component is then scanned with a continuous path, named contour. This leads to different parameters for the optimization of the surface: for the horizontal surfaces, the hatch distance $d_{H}$ can be varied in addition to the laser power *P* and the scan speed *v*. If the parameters for the uppermost layers, i.e., for visible surfaces instead of the interior, are changed, this surface is called upskin. For the lateral, vertical surfaces (contour), the distance between hatch and contour $d_{O}$ is varied instead of the hatch distance. The layer height *t* also has an influence on the contour’s roughness, yet in this paper it has been kept at a constant value of 50 μm to keep the number of variables low. The same applies to the spot diameter $d_{S}$, which was maintained at a constant value of 200 μm. The exposure strategy and process parameters are shown in Figure 3.

The parameters mentioned above (*P*, *v*, $d_{H}$ and $d_{O}$) were varied with three different levels each for the upskin and contour surfaces. The individual values for each level can be found in Table 1. Often, the energy density is used as a parameter to quantify the energy input of the laser into the material. The energy density results from the quantities varied in this study according to the following formula for hatched surfaces [10]:

(1) $$\rho_{E}=\frac{P}{v\;\cdot \;d_{H}\;\cdot \;t}\;\left(J/{\mathrm{mm}}^{3}\right).$$

In the case of single scanning tracks, such as in contour exposure, the spot diameter $d_{S}$ is used instead of the hatch distance $d_{H}$ and the layer thickness *t* [8]:

(2) $$\rho_{E,S}=\frac{P}{v\;\cdot \;d_{S}}\;\left(J/{\mathrm{mm}}^{2}\right).$$

In addition to surface roughness, the parameters varied have a significant influence on density as well. Since the focus of this paper is on the surfaces, the exposure strategy inside the part has not been changed. For the interior of the part, the manufacturer’s settings were used, which are close to the results presented by Mutua et al. [10] for maximum density.

The material used in this study is a metal powder similar in composition to 1.2709, commercialized by the machine supplier as *Maraging II*, which is manufactured by *Sandvik Osprey* (Neath, UK). The chemical composition can be found in Table 2. The particle size distribution is in the range of 20–45 μm [17].

During the SLM and milling process, the build plate temperature is set to 50 ${}^{\circ }C$, and the residual oxygen content is within a range of 1–2% in nitrogen atmosphere.

The test specimens are cuboids with the dimensions 10×10×12 ${\mathrm{mm}}^{3}$, built directly onto the build plate. After sawing off with a band saw, cube-shaped specimens with an edge length of about 10 mm were obtained. Since the different variants for the contour surfaces as well as for the upskin surfaces can be combined in one test specimen, nine specimens and a reference with the manufacturer’s settings were fabricated. The number of samples is a result of the Taguchi methodology, which is described in more detail in Section 2.3.

#### 2.1.3. Milling

**Figure 4:**
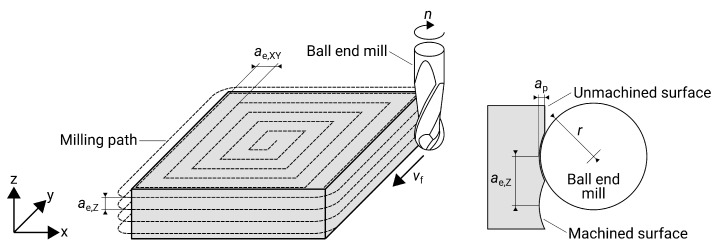
Milling strategy and parameters for a ball end mill.

For milling in general and ball end milling in particular, several parameters have a decisive influence on the surface roughness of the component being machined. In previous studies, cutting speed $v_{c}$, feed per tooth $f_{z}$, and radial depth of cut $a_{e}$ have been proven to be the most important influencing parameters [14]. As this work deals with surface quality and not wear, tool life or productivity, only finishing is considered. Therefore, the axial depth of cut $a_{p}$ resulting from the remaining stock of the previous roughing operation is kept constant. Figure 4 shows the milling strategy used in this study and the corresponding parameters. The typical input parameters for milling machines, spindle speed *n*, and feed rate $v_{f}$, are obtained in accordance with the following formulas using tool diameter *d* and number of teeth *z* [18]:

(3) $$\begin{array}{c} n=\frac{v_{c}}{\pi \;\cdot \;d}\;\left(\mathrm{rpm}\right), \end{array}$$

(4) $$\begin{array}{c} v_{f}=z\;\cdot \;f_{z}\;\cdot \;n\;\left(\mathrm{mm}/\min\right). \end{array}$$

As before, three factors with three levels each were selected. The different factors and the corresponding levels are summarized in Table 3.

For finishing, a carbide ball end mill with a diameter of 2 mm respectively a radius *r* of 1 mm and a geometry specially designed for this process (undercut, see Figure 2) was used (*Mitsubishi MS2XLB*, Tokyo, Japan). It should be noted that the previous roughing operation was carried out with a 2 mm milling cutter (*Mitsubishi VF2XLB*) with *n* = 40,000 rpm, $v_{f}=2000$ mm/min, $a_{e}=0.15$ mm, $a_{p}=0.22$ mm, respectively. The process conditions during milling regarding temperature and atmosphere do not differ from those during SLM.

The sample dimensions are 10×10×10 ${\mathrm{mm}}^{3}$ after removal from the build plate, as for the SLM components in the previous section. Again, nine specimens, as a result due to the DoE described in Section 2.3, and a reference specimen with manufacturer’s settings, were fabricated.

### 2.2. Measurements

For surface characterization, 3D profilometry has been found to be a suitable method for AM components [19]. Although profile roughness $R_{a}$ is currently the most commonly used parameter, it is recommended to use areal roughness $S_{a}$ as a parameter because it is more significant since data are also obtained in the lateral direction [20,21]. The surface texture parameter $S_{a}$ represents the arithmetical mean height of a surface and is described by the following equation, which is specified in DIN EN ISO 25178 [22]:

(5) $$S_{a}=\frac{1}{A}\underset{A}{\;\int \int \;}\left|z\left(x,y\right)\right|\;\mathrm{dx}\;\mathrm{dy}\;\left(\mu m\right).$$

The areal surface roughness of unmachined SLM parts was measured using a macroscope (*Keyence VR-3200*, Osaka, Japan) employing structured light to gain 3D topography data. An area of 9×9 ${\mathrm{mm}}^{3}$ was evaluated, i.e., the specimen’s edge is not included in the evaluation. One assembled image consists of 20 single captures, each taken at 80× magnification. The image has been corrected for plane tilt. The S-Filter was set to 12 μm due to the macroscope’s areal resolution and an L-Filter of 2.5 mm was chosen in accordance to the results of Triantaphyllou et al. [19]. Two adjacent surfaces were selected from the four vertical surfaces and the mean $S_{a}$ was calculated. For the milled specimens, the areal surface roughness was measured using an optical microscope (*Bruker Contour GT-K*, Billerica, USA) employing white light interferometry and *Vision 64* software. In this case, the examined area is 1×1 ${\mathrm{mm}}^{3}$ in size. Again, several pictures were assembled and a correction for plane tilt was made. One stitched image consists of six images, each taken at 11× effective magnification. For these samples, the S-Filter was set to 2.5 μm due to the lateral resolution and an L-Filter of 0.25 mm was set. Due to the consistent surface quality compared to unmachined parts, only one surface of the specimen’s vertical faces was measured.

### 2.3. Statistical Analysis and Optimization

Unal and Dean describe the process of an experiment conducted using DoE and the Taguchi method [13].

**Figure 5:**
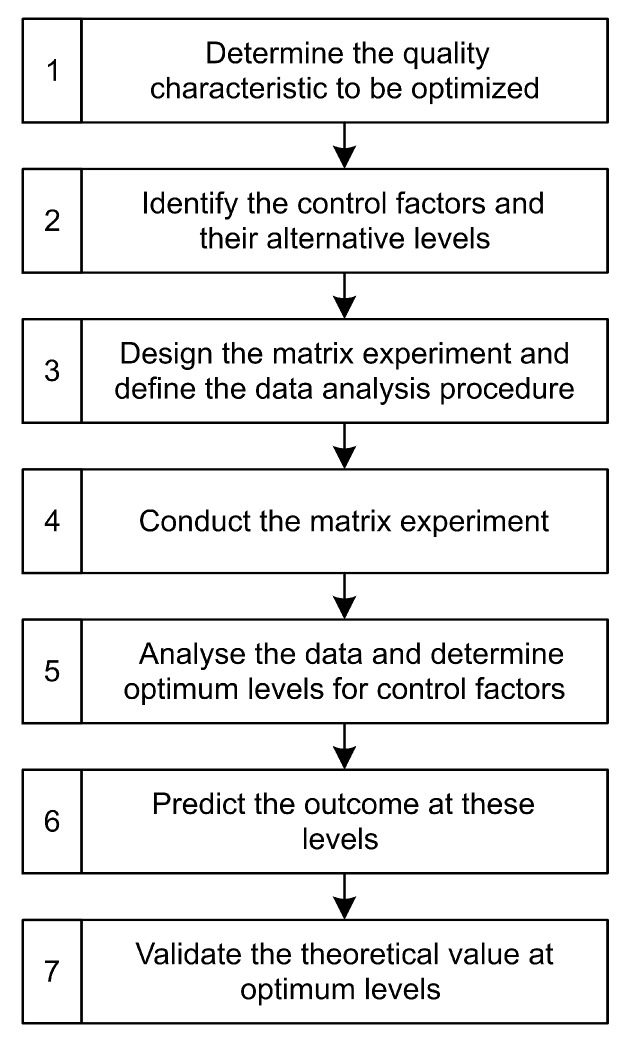
Flowchart of the tasks carried out in this paper according to Unal and Dean [13].

Based on this, the procedure of statistical optimization starts with the determination of the quality characteristic (see Figure 5). The average surface roughness $S_{a}$ in μm is the indicator that is measured and evaluated. The next step is to define the control factors, i.e., the parameters, and their levels. These factors and levels were determined for SLM and milling in the previous section. Three factors with three levels each were chosen for all series of experiments. This is followed by designing the matrix experiment. A full factorial design would result in $3^{3}=27$ tests each. The Taguchi method uses an L9 orthogonal array for this set of factors and levels, which involves only nine experiments [23]. The reduction in effort becomes evident at this point. After the experiments have been conducted, the signal-to-noise ratio (S/N ratio) is used as a tool to evaluate the results. Since the goal of the optimization is a lowest possible $S_{a}$-value, it is a “the-smaller-the-better problem”. The S/N ratio $\eta_{i}$ in dB is then calculated using the following formula with the measured value $y_{i}$ and the number of repetitions *n* [23]:

(6) $$\eta_{i}=-10\;\cdot \;\log_{10}\left(\frac{1}{n}\sum_{i=1}^{n}{y_{i}}^{2}\right)\;\left(\mathrm{dB}\right).$$

The influence of the individual factors (known as effect) can then be calculated using an analysis of means (ANOM) from the S/N ratio results [23]. For this purpose, the statistics software *Minitab 18* was used in this study. The statistically best value results from the combination of the three best levels for each factor. This value can be predicted using regression. A simple linear regression is a proven approach and can be expressed by formula (7) given below with the determined value $\hat{y}$, coefficients *b* and factor levels *x* [23]:

(7) $$\hat{y}=b_{0}+b_{1}\;\cdot \;x_{1}+b_{2}\;\cdot \;x_{2}+b_{2}\;\cdot \;x_{2}.$$

The result of the regression can then be validated by a final experiment to confirm the optimal value resulting from the respective best factor levels.

## 3. Experimental Results and Discussion

Four test series were evaluated, since the unmachined and milled components were each measured on the vertical and horizontal surfaces.

### 3.1. Selective Laser Melting

**Figure 6:**
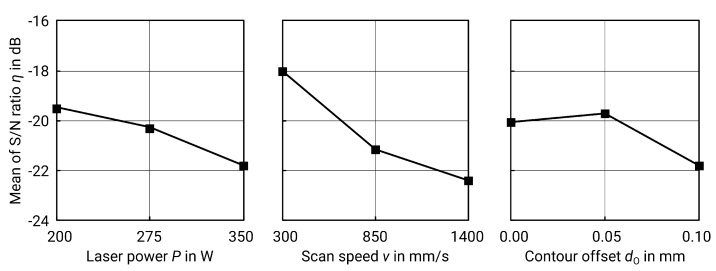
Main effect plot for S/N ratios for $S_{a}$ of contour surfaces.

For vertical surfaces, i.e., contours, the performed test combinations, the respective energy density and the corresponding results can be found in Table 4. The *p*-value of the ANOVA (see Table 5) shows that the scan speed *v* is the most important factor. The delta S/N value (spreading of S/N values, see Table 6 and Figure 6) confirms this because the larger the spread, the stronger the influence of the factor.

The best result of the test series is specimen no. 1 (see Table 4) with an obtained roughness $S_{a}$ of 6.5 μm. However, the individual best factor levels result in a theoretical optimum at P=200W, v=300mm/s and $d_{O}=0.05\mathrm{mm}/s$. The resulting roughness from these factor levels can be predicted using linear regression using formula (8) and results in $\hat{S_{a}}=7.2\;\mu m$:

(8) $$\begin{array}{c} \hat{S_{a}}=1.58+0.01667\;\cdot \;P+0.00455\;\cdot \;v+18.33\;\cdot \;d_{O}\;\left(\mu m\right),\\ R^{2}=89.94\%. \end{array}$$

In order to validate the result, a further test specimen was produced and tested with the optimum factor levels. The $S_{a}$ measured is 7.50 μm which is in good agreement with the afore predicted value. However, both the calculated and the measured $S_{a}$ are not the best values of the test series. In order to achieve the lowest possible roughness, the factor levels from experiment number one should therefore be used (see Table 4). The only difference between the parameters is the contour offset $d_{O}$, which is zero in the actual optimum. An interpretation of the statistical data supports the assumption that $d_{O}$ can be set to zero: The *p*-value of ANOVA shows that $d_{O}$ is the least significant parameter. Abele and Kniepkamp confirm the assumption that $d_{O}$ can be neglected, either due to the dominant effect of energy or due to insufficient spread of the design space [5]. This shows that the model is a very good approximation but is also accompanied by certain errors, though only in the range of one micron.

**Figure 7:**
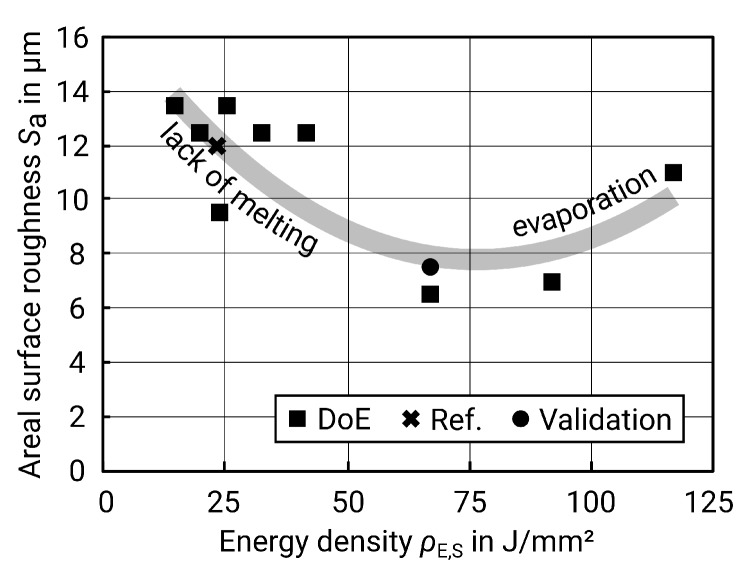
Surface roughness $S_{a}$ versus energy density $\rho_{E,S}$ for contour surfaces (including nine values as a result of the design of experiments (DoE), one value as a reference (see Table 4), and one value from the validation; grey line for clarification purposes only).

When considering energy density, the result meets the expectations as well: too low energy density leads to an instable track with lack of melting and balling and thus increases the roughness [5,24]. On the other hand, too much energy causes evaporation and also leads to high roughness [25]. Figure 7 below illustrates this relationship.

**Figure 8:**
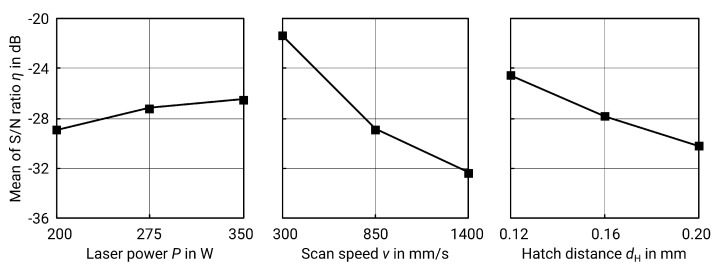
Main effect plot for S/N ratios for $S_{a}$ of upskin surfaces.

Upskin surfaces are presented and evaluated in the same way as contour surfaces. The experiments conducted, energy density, and measurement results can be found in Table 7. Again, the *p*-value of the ANOVA (see Table 8) and the delta S/N-value (see Table 9 and Figure 8) show that the scanning speed *v* is the most significant factor.

The best results of the test series are no. 1 and no. 4 (see Table 7) with P=200W and 275 W, v=300mm/s and $d_{H}=0.12\;mm$. The best levels for each factor, following the respective maximum S/N-value, result in a theoretical optimum for $S_{a}$ at P=350W, v=300mm/s and $d_{H}=0.12\;mm$.

The $S_{a}$-value for these factor levels can be predicted using regression. However, a simple linear regression might cause negative values in this case. Since this is physically impossible for surface roughness values, the model must be modified. A quadratic transformation was performed for this purpose, resulting in the following equation:

(9) $$\begin{array}{c} \sqrt{\hat{S_{a}}}=-0.00441\;\cdot \;P+0.00286\;\cdot \;v+24.10\;\cdot \;d_{H}\;\left(\mu m\right),\\ R^{2}=99.62\%. \end{array}$$

The predicted roughness for the combination of factor levels is $\hat{S_{a}}=4.87\;\mu m$. To validate the prediction and the previous optimization, another test specimen was produced. The roughness $S_{a}$ determined in this case is 9 μm and thus lower than in all previous tests, even if there is a deviation from the prediction. The prediction by regression should, however, be considered carefully. If a logarithmic transformation is chosen instead of a quadratic transformation, the fit is not as accurate ($R^{2}=93.16\%$). However, the predicted value is closer to reality ($\hat{S_{a}}=7.93\;\mu m$). Hence, a validation is essential.

**Figure 9:**
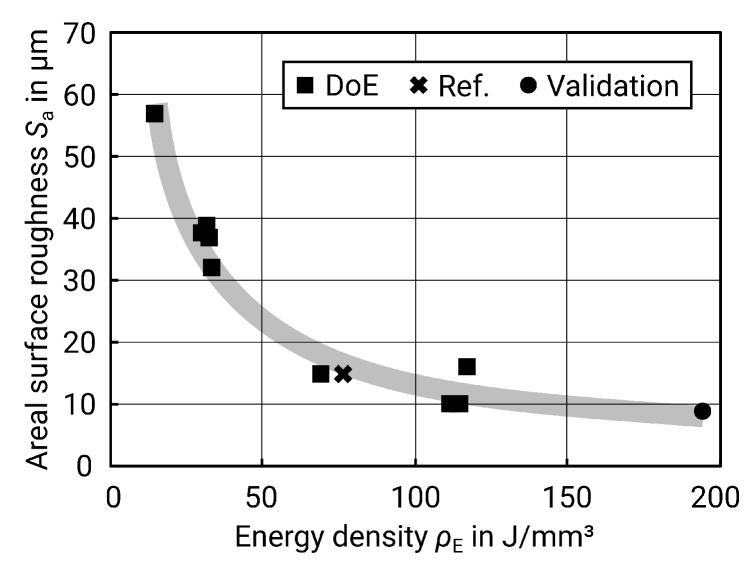
Surface roughness $S_{a}$ plotted versus energy density $\rho_{E}$ for upskin surfaces (including nine values as a result of the DoE, one value as a reference (see Table 7) and one value from the validation; grey line for clarification purposes only).

Regarding the roughness as a function of the energy density, the same effects occur as on the contour surfaces. A certain energy density is necessary to fully melt a continuous track, but, if the energy is too high, evaporation occurs [5,24,25]. However, this area of too much energy did not become evident in the tests carried out, as Figure 9 shows. Due to the underlying layers of solidified material, the heat is dissipated better at the upskin surfaces, which is why the region of evaporation is not reached here. On the contour surfaces, however, a larger amount of heat is dissipated by the surrounding powder bed, which conducts heat less well than solid material. A further increase of the energy density, however, may not be appropriate, as the energy input would otherwise be too high and the surface roughness would increase again due to evaporation.

A high energy density is helpful in achieving low surface roughness. Due to the resulting higher temperatures, the melt pool has more time to flatten during cooling. In addition, a low hatch distance, which also increases the energy density according to formula (1), leads to a high overlap between the melt paths. This results in fewer peaks and valleys, which smoothens the surface as well [24]. The importance of the hatch distance on roughness is also evident from the ANOVA, where the *p*-value of $h_{D}$ ranks second.

Table 10 summarizes the optimized values for both the contour and upskin surfaces produced by SLM.

### 3.2. Milling

**Figure 10:**
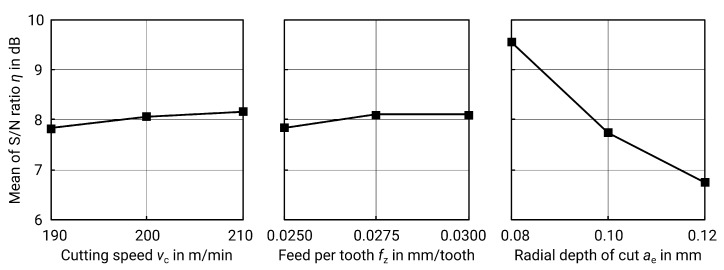
Main effect plot for S/N ratios for $S_{a}$ of milled vertical surfaces.

The combinations of the various factor levels for ball end milling of vertical surfaces can be found in Table 11. In addition, the spindle speed *n* and the feed rate $v_{f}$, calculated using formulas (3) and (4), are given. For milling of the vertical side surfaces, both the *p*-value of ANOVA (see Table 12) and delta S/N (see Table 13 and Figure 10) show that the radial depth of cut $a_{e}$ is the most important factor.

Based on the best factor levels, a theoretical optimum is obtained which corresponds to a test already conducted. The best surface quality results from the combination of cutting speed $v_{c}=210$ m/min, $f_{z}=0.0275$ mm/tooth and $a_{e}=0.08$ mm, corresponding to specimen no. 8 with a surface roughness $S_{a}$ of 0.327 μm. In machining processes, however, the line roughness is usually specified. For this reason, these values are given as well. For these factor levels, the corresponding $R_{a}$ is 0.292 μm and $R_{z}$ is 1.943 μm (cut off $\lambda_{C}=0.25$ mm, $\lambda_{S}=2.5$ μm). The results from measurement and statistics meet the expectations and results of previous work: on one hand, a high cutting speed leads to better surfaces; on the other hand, the radial depth of cut is the most important parameter [14]. The influence of the depth of cut can be explained by the geometry of the milling cutter and the z-constant milling process. The result is an undulating surface that is mainly dependent on radial depth of cut and the radius of the milling cutter (see Figure 4). According to Peterka, the theoretical roughness $R_{a,\mathrm{calc}}$ for the specified parameters is calculated according to the formulae below [26]:

(10) $$\begin{array}{c} R_{a,\mathrm{calc}}=\frac{r^{2}}{a_{e}}\left(2\phi_{z}-\sin2\phi_{z}\right)\;\cdot \;1000\;\left(\mu m\right), \end{array}$$

(11) $$\begin{array}{c} \phi_{z}=\arccos\left(\frac{1}{2}\cos\beta +\frac{r}{a_{e}}\beta \right)\;\left(\mathrm{rad}\right), \end{array}$$

(12) $$\begin{array}{c} \beta =\arcsin\left(\frac{a_{e}}{2r}\right)\;\left(\mathrm{rad}.\right) \end{array}$$

**Figure 11:**
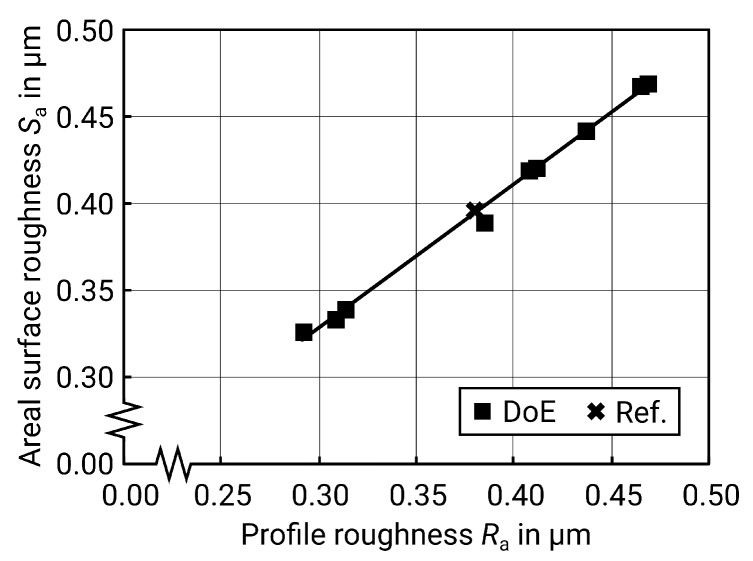
Linear regression of $S_{a}\left(R_{a}\right)$ for vertical milled surfaces.

For tool radius r=1 mm and $a_{e}=0.08$ mm, a $R_{a,\mathrm{calc}}=0.205$ μm is obtained. As Harcarik and Jankovych describe, this value for profile roughness $R_{a}$ can be converted to surface roughness $S_{a}$ using a coefficient resulting from linear regression (see Figure 11) and the formula below [20]:

(13) $$\begin{array}{c} {\hat{S_{a}}}_{,\mathrm{calc}}=0.8321\;\cdot \;R_{a,\mathrm{calc}}+0.0785\;\left(\mu m\right),\\ R^{2}=99.48\%. \end{array}$$

This results in ${\hat{S_{a}}}_{,\mathrm{calc}}=0.249\;\mu m$. The deviation between measured and calculated values may result from a process- and machine-dependent coefficient, since, for example, the rigidity of the machine and the workpiece need to be taken into account as well. It can therefore be assumed that the optimum is reached at this point with the given factor levels.

Since the optimal combination of factor levels has already been evaluated, in this case, a regression to predict the roughness from all three factor levels and a further validation can be omitted.

**Figure 12:**
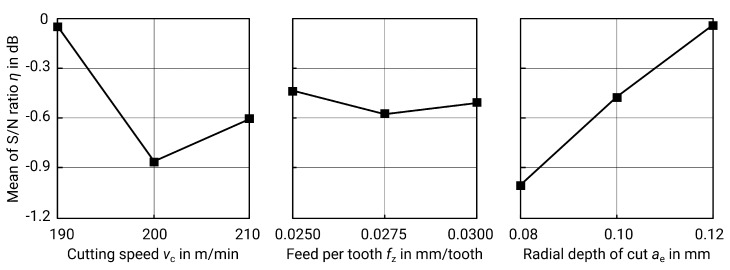
Main effect plot for S/N ratios for $S_{a}$ of milled horizontal surfaces.

The examination of the horizontal milled surfaces shows considerable differences. The respective variations and their corresponding surface roughness values can be found in Table 14. Once again, the most significant factor is the radial depth of cut $a_{e}$, according to the *p*-value from the ANOVA in Table 15 and the spread of the S/N values from Table 16 and Figure 12.

However, the curve of the mean S/N values is contrary to the expectations. Due to the geometric relationship between the roughness and the depth of cut shown above, a minimum $a_{e}$ as an optimum should also be expected here. The statistical analysis reveals though that a high value for $a_{e}$ seems to be desirable. Thus, the statistics do not correspond to the real behaviour to be expected. Since the ANOVA shows that $a_{e}$ is the only significant parameter, and assuming that the actual roughness $S_{a}$ results from the theoretical roughness ${\hat{S_{a}}}_{,\mathrm{calc}}$ and a process- and machine-specific coefficient, this means that other factors play a role in milling of horizontal surfaces with a ball end mill and the parameters examined in this paper. This can include, for example, the rigidity of the machine, the tool, and the workpiece. The reliability of the statistics is therefore limited in this case.

The best surface roughness is obtained for test number 3 with cutting speed $v_{c}=190$ m/min, $f_{z}=0.03$ mm/tooth and $a_{e}=0.12$ mm, resulting in $S_{a}=0.964\;\mu m$. Using the best factor levels in each case, a theoretical optimum is obtained at $v_{c}=190$ m/min, $f_{z}=0.025$ mm/tooth and $a_{e}=0.12$ mm.

The linear regression, which results according to formula (14), also reflects the insufficient predictability, since $R^{2}$ is only 73.04%. An adjustment of the regression model towards a higher $R^{2}$ is possible, but this does not change the physically difficult circumstances of the milling process:

(14) $$\begin{array}{c} \hat{S_{a}}=0.62+0.00337\;\cdot \;v_{c}+2.33\;\cdot \;f_{z}-2.958\;\cdot \;a_{e}\;\left(\mu m\right),\\ R^{2}=73.04\%. \end{array}$$

In order to evaluate the result with the optimal factor levels, another specimen was produced. The measured surface roughness for the optimum factor levels is $S_{a}=0.835\;\mu m$ and so it is lower than the previously measured values and also close to the value obtained by regression of $\hat{S_{a}}=0.963\;\mu m$, but these values must be viewed critically.

This is because milling the horizontal surfaces represents the greatest challenge for ball end milling on a machine with three axes. In this case, the central cutting edge of the milling cutter is used. However, this part of the cutting edge has no velocity because it is in the axis of rotation. This means that the material is deformed rather than cut in this area and the surface quality deteriorates. In addition, it is to be expected that the surface quality increases with the cutting speed. However, the actual cutting speed is lower than the set speed because it is calculated using the larger outer radius instead of the radius actually used.

An increasing radial depth of cut reduces the number of toolpaths in which the material is deformed instead of milled. In addition, a larger amount of the cutter’s radius is engaged, which means that the actual cutting speed is higher. Thus, when ball-milling horizontal surfaces, in the given range of values, a larger $a_{e}$ may lead to a better surface quality. However, this relationship is only valid within the range examined in this paper because above a certain $a_{e}$ the influence of geometry becomes dominant due to the z-constant milling strategy with the ball end mill, which leads to an undulating surface.

A further improvement may possibly be achieved with a different type of end mill. However, a variation of the milling tools is beyond the scope of this work and the selected range of parameters for optimization.

**Figure 13:**
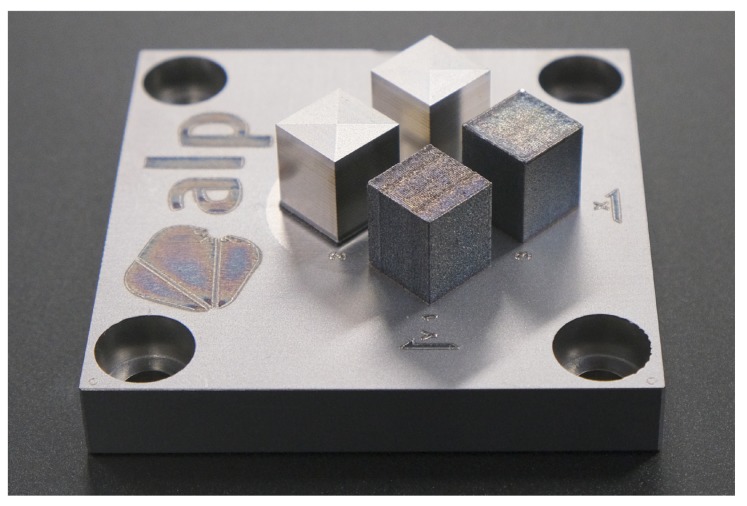
Image showing the optimized surfaces in comparison to the surfaces obtained with the manufacturer’s settings (back row: milled; front row: unmachined/SLM; each left: reference; on the right: optimized surface).

Table 17 summarizes the optimized values for both the vertical and horizontal surfaces produced by milling in the hybrid AM process. The photograph in Figure 13 shows the different surfaces that have been achieved, with the unmachined surfaces in particular showing visible differences.

## 4. Conclusions

In this paper, a hybrid manufacturing process comprising selective laser melting and three-axis milling with the aim of optimizing surface roughness $S_{a}$ was investigated. A distinction was made between vertical surfaces (also known as contour in SLM) and horizontal surfaces (called hatch in SLM). This results in four different processes, which have all been statistically optimized using DoE and the Taguchi method.

When optimizing the SLM process, laser power, scanning speed, and hatch distance have been varied. The results were also discussed in terms of energy input, respectively the energy density. The optimal combination of these parameters was both predicted by regression and validated in an experiment. By selecting optimal factor levels, it was possible to reduce the roughness for upskin surfaces to $S_{a}=9.0\;\mu m$, which corresponds to a reduction of 40% as compared to manufacturer’s recommendations. The roughness of contour surfaces could be reduced by 37.5% to $S_{a}=7.5\;\mu m$.

To optimize the milling process, cutting speed, feed per tooth, and radial depth of cut have been varied. Here, the results were compared with theoretical values for roughness resulting from geometrical relationships. In this case, the surface roughness of vertical surfaces, which was already satisfactory before optimization, was even further reduced to $S_{a}=0.397\;\mu m$, corresponding to a reduction of 17.6%.

Milling horizontal surfaces has proven to be a challenge due to the use of ball end mills. The surface roughness could also be reduced by 23.1% to $S_{a}=0.835\;\mu m$ using statistical methods.

Overall, it is shown that the Taguchi method is well suitable for the optimization of hybrid additive manufacturing using SLM and milling. By using DoE, the experimental effort can be reduced, which benefits complex and costly processes such as additive manufacturing. However, a critical evaluation of the results from statistics and measurement is essential. The improved surface quality obtained through this work makes a wider use of this hybrid manufacturing process possible, as the improved surfaces may open up new fields of application.

## Figures and Tables

**Table 1:** Manufacturer’s settings as a reference and different levels for optimization of SLM parts made of 1.2709. Note that the manufacturer does not make any distinction between the interior (hatch) and upskin surfaces.

Parameter	Reference Hatch	Reference Contour	Level 1	Level 2	Level 3
Laser Power *P* (W)	320	320	200	275	350
Scan speed *v* (mm/s)	700	1400	300	850	1400
Contour offset $d_{O}$ (mm)	-	0	0	0.05	0.10
Hatch distance $d_{H}$ (mm)	0.12	-	0.12	0.16	0.20
Spot diameter $d_{S}$ (μm)	200 (const.)				
Layer height *t* (μm)	50 (const.)				

**Table 2:** Chemical composition of the steel powder used in this study in percentage by mass (wt%) per element according to [17].

Element	Fe	Ni	Co	Mo	Ti	Cr	Mn	Si	Al	C	S
wt%	Balance	17–19	8.5–9	4.5–5.2	0.6–0.8	≤0.3	≤0.1	≤0.1	0.05–0.15	≤0.03	≤0.01

**Table 3:** Manufacturer’s settings for finishing as a reference and different levels for input parameters.

Parameter	Reference	Level 1	Level 2	Level 3
Cutting speed $v_{c}$ (m/min)	189	190	200	210
Feed per tooth $f_{z}$ (mm/tooth)	0.0267	0.0250	0.0275	0.0300
Radial depth of cut $a_{e}$ (mm)	0.10	0.08	0.10	0.12
Axial depth of cut $a_{p}$ (mm)	0.03 (const.)			

**Table 4:** Factors and their respective levels for the nine experiments regarding contour and reference with manufacturers’ settings.

	*P* (W)	*v* (mm/s)	*d*_O_ (mm)	*ρ_E,S_* (J/mm^2^)	*S*_a_ (μm)	*η* (dB)
1	200	300	0.00	66.67	6.50	−16.26
2	200	850	0.05	23.53	9.50	−19.55
3	200	1400	0.10	14.29	13.50	−22.61
4	275	300	0.05	91.67	7.00	−16.90
5	275	850	0.10	32.35	12.50	−21.94
6	275	1400	0.00	19.64	12.50	−21.94
7	350	300	0.10	116.67	11.00	−20.83
8	350	850	0.00	41.18	12.50	−21.94
9	350	1400	0.05	25.00	13.50	−22.61
Ref.	320	1400	0.00	22.86	12.00	−21.61

**Table 5:** Analysis of variance for S/N ratios of vertical surfaces (contour), $R^{2}=99.18\%$.

Source	*df*	Sum of Squares	*F*	*p*
*P*	2	8.34	21.56	0.044
*v*	2	30.70	79.39	0.012
$d_{O}$	2	7.60	19.65	0.048
Residual Error	2	0.39		
Total	8	47.02		

**Table 6:** Average S/N ratios for vertical surfaces (contour).

Level	*p*	*v*	*d*_O_
1	−19.47	−18.00	−20.04
2	−20.26	−21.14	−19.69
3	−21.79	−22.38	−21.79
Delta	2.32	4.39	2.1
Rank	2	1	3

**Table 7:** Factors and their respective levels for the experiments regarding upskin and reference with manufacturers’ settings.

	*P* (W)	*v* (mm/s)	*d*_H_ (mm)	*ρ_E_* (J/mm^2^)	*S*_a_ (μm)	*η* (dB)
1	200	300	0.12	111.11	10.00	−20.00
2	200	850	0.16	29.41	38.00	−31.60
3	200	1400	0.20	14.29	57.00	−35.12
4	275	300	0.16	114.58	10.00	−20.00
5	275	850	0.20	32.35	37.00	−31.36
6	275	1400	0.12	32.74	32.00	−30.10
7	350	300	0.20	116.67	16.00	−24.08
8	350	850	0.12	68.63	15.00	−23.52
9	350	1400	0.16	31.25	39.00	−31.82
Ref.	320	700	0.12	76.19	15.00	−23.52

**Table 8:** Analysis of variance for S/N ratios of upskin surfaces, $R^{2}=96.59\%$.

Source	*df*	Sum of Squares	*F*	*p*
*P*	2	9.42	1.08	0.480
*v*	2	188.84	21.68	0.044
$d_{H}$	2	48.21	5.53	0.153
Residual Error	2	8.71		
Total	8	255.19		

**Table 9:** Average S/N ratios for upskin surfaces.

Level	*P*	*v*	*d*_H_
1	−28.90	−21.36	−24.54
2	−27.16	−28.83	−27.81
3	−26.48	−32.35	−30.19
Delta	2.43	10.99	5.65
Rank	3	1	2

**Table 10:** Optimized factor levels for SLM-made surfaces.

	*P* (W)	*v* (mm/s)	*d*_O_ (mm)	*d*_H_ (mm)	*S*_a_ (μm)
Contour	200	300	0.00	-	6.50
Upskin	350	300	-	0.12	9.00

**Table 11:** Factors and their respective levels for ball end milling of vertical surfaces, $R_{a}$ provided additionally ($\lambda_{C}=0.25$ mm, $\lambda_{S}=2.5$ μm).

	*v*_c_ (m/min)	*f*_z_ (mm/tooth)	*a*_e_ (mm)	*n* (min^−1^)	*v*_f_ (mm/min)	*R*_a_ (μm)	*S*_a_ (μm)	*η* (dB)
1	190	0.0250	0.08	30,239	1512	0.314	0.339	9.396
2	190	0.0275	0.10	30,239	1663	0.411	0.421	7.514
3	190	0.0300	0.12	30,239	1814	0.469	0.469	6.577
4	200	0.0250	0.10	31,831	1592	0.407	0.419	7.556
5	200	0.0275	0.12	31,831	1751	0.437	0.442	7.092
6	200	0.0300	0.08	31,831	1910	0.309	0.333	9.551
7	210	0.0250	0.12	33,423	1671	0.465	0.468	6.595
8	210	0.0275	0.08	33,423	1838	0.292	0.327	9.709
9	210	0.0300	0.10	33,423	2005	0.385	0.390	8.179
Ref.	189	0.0267	0.10	30,080	1606	0.380	0.397	8.024

**Table 12:** Analysis of variance for S/N ratios of ball end milled vertical surfaces, $R^{2}=98.47\%$.

Source	*df*	Sum of Squares	*F*	*p*
$v_{c}$	2	0.18	0.92	0.522
$f_{z}$	2	0.13	0.68	0.596
$a_{e}$	2	12.07	62.97	0.016
Residual Error	2	0.19		
Total	8	12.56		

**Table 13:** Average S/N ratios for ball end milled vertical surfaces.

Level	*v*_c_	*f*_z_	*a*_e_
1	7.829	7.849	9.552
2	8.066	8.105	7.750
3	8.161	8.102	6.754
Delta	0.332	0.256	2.798
Rank	2	3	1

**Table 14:** Factors and their respective levels for ball end milling of horizontal surfaces, $R_{a}$ provided additionally ($\lambda_{C}=0.25$ mm, $\lambda_{S}=2.5$ μm).

	*v*_c_ (m/min)	*f*_z_ (mm/tooth)	*a*_e_ (mm)	*n* (min^−1^)	*v*_f_ (mm/min)	*R*_a_ (μm)	*S*_a_ (μm)	*η* (dB)
1	190	0.0250	0.08	30,239	1512	0.887	1.042	−0.357
2	190	0.0275	0.10	30,239	1663	0.823	1.013	−0.112
3	190	0.0300	0.12	30,239	1814	0.763	0.964	0.318
4	200	0.0250	0.10	31,831	1592	0.923	1.103	−0.852
5	200	0.0275	0.12	31,831	1751	0.841	1.041	−0.349
6	200	0.0300	0.08	31,831	1910	0.972	1.173	−1.386
7	210	0.0250	0.12	33,423	1671	0.870	1.011	−0.095
8	210	0.0275	0.08	33,423	1838	0.919	1.156	−1.259
9	210	0.0300	0.10	33,423	2005	0.890	1.054	−0.457
Ref.	189	0.0267	0.10	30,080	1606	0.898	1.086	−0.717

**Table 15:** Analysis of variance for S/N ratios of ball end milled horizontal surfaces, $R^{2}=97.26\%$.

Source	*d*_f_	Sum of Squares	*F*	*p*
$v_{c}$	2	1.03	14.98	0.063
$f_{z}$	2	0.03	0.42	0.704
$a_{e}$	2	1.38	20.08	0.047
Residual Error	2	0.07		
Total	8	2.51		

**Table 16:** Average S/N ratios for ball end milled horizontal surfaces.

Level	*v*_c_	*f*_z_	*a*_e_
1	−0.050	−0.435	−1.001
2	−0.862	−0.573	−0.474
3	−0.604	−0.508	−0.042
Delta	0.812	0.139	0.959
Rank	2	3	1

**Table 17:** Optimized factor levels for milling of vertical and horizontal surfaces.

	*v*_c_ (m/min)	*f*_z_ (mm/tooth)	*a*_e_ (mm)	*S*_a_ (μm)
Vertical	210	0.0275	0.08	0.327
Horizontal	190	0.0250	0.12	0.835
